# Fabrication and spectroscopic studies on highly luminescent CdSe/CdS nanorod polymer composites

**DOI:** 10.3762/bjnano.1.11

**Published:** 2010-11-29

**Authors:** Jana Bomm, Andreas Büchtemann, Angela Fiore, Liberato Manna, James H Nelson, Diana Hill, Wilfried G J H M van Sark

**Affiliations:** 1 Fraunhofer Institute for Applied Polymer Research (IAP), Geiselbergstraße 69, 14476 Potsdam-Golm, Germany; 2 National Nanotechnology Laboratory of INFM (NNL), DistrettoTecnologico ISUFI Via, Arnesano, 73100 Lecce, Italy; 3 3Lawrence Berkeley National Laboratory and Department of Chemistry, University of California, Berkeley, California 94720, United States; 4 Department of Chemistry, University of Potsdam, Karl-Liebknecht-Str. 24-25, 14476 Potsdam-Golm, Germany; 5 Science, Technology and Society, Copernicus Institute of Sustainable Development and Innovation, Utrecht University, Heidelberglaan 2, 3584 CS Utrecht, the Netherlands

**Keywords:** CdSe, luminescence lifetime, nanocomposites, nanorods, quantum yield

## Abstract

Highly luminescent nanocomposites were prepared by incorporating CdSe/CdS core/shell nanorods into different polymer matrices. The resulting nanocomposites show high transparency of up to 93%. A photoluminescence quantum efficiency of 70% was obtained, with an optimum combination of nanorod (0.05 wt %) and at a UV-initiator concentration of 0.1 wt % for poly(lauryl methacrylate). Nanorods tend to agglomerate in cellulose triacetate.

## Introduction

Semiconductor nanoparticles have attracted great interest in recent years because of their fascinating optical properties. Their emission wavelength can be tuned directly by changing their size and shape as a result of quantum confinement effects. By reducing the particle size, the band-gap increases and the emitted light is blue-shifted [1–2]. In contrast to spherical quantum dots (QDs), nanorods (NRs) show anisotropic properties such as linearly polarized absorption, emission and lasing, and thus have potential applications in a new generation of light-emitting diodes (LEDs), lasers or luminescent solar concentrators [3–6]. One of the challenges of these applications is the incorporation of inorganic nanoparticles into organic polymer matrices, since this is usually accompanied by phase separation, aggregation of nanoparticles, loss of transparency and luminescence quenching due to exciton energy transfer [2,7]. Several methods have been described to overcome these problems for quantum dot polymer composites. For example, Bawendi and coworkers incorporated trioctylphosphine oxide covered CdSe/ZnS QDs in poly(lauryl methacrylate) (PLMA) [6,8], while Woelfle and Claus dispersed CdSe/ZnS QDs in an ionic liquid that was compatible with poly(methyl methacrylate) (PMMA) [6]. However, in both these cases chemical attack of QDs by initiator radicals produced from azobisisobutyronitrile (AIBN) during the thermal polymerization process leads to luminescence quenching and as a result nanocomposites with photoluminescence (PL) quantum efficiency (QE) of less than 40% were obtained [6–8].

**Figure 1:**
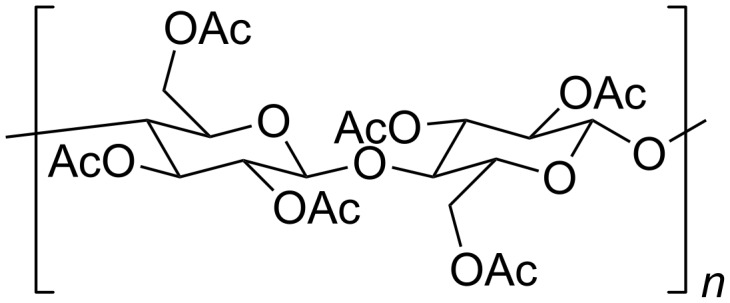
Structure of CTA.

Here we present two different methods to fabricate nanorod polymer composites: (a) UV-polymerization and (b) a radical free drop-casting process. Core/shell NRs were chosen as they exhibit higher stability against photooxidation and chemical attack by radicals [2,9]. The band-gap within a type-I core/shell nanocrystal gradually increases from the inside to the outside [2,10]. In our first method, where radicals are present, the NRs were dispersed at several concentrations in a monomer mixture of lauryl methacrylate (LMA) with the cross-linking agent ethylene glycol dimethacrylate (EGDM) and the liquid UV-initiator Darocure^®^ 4265. LMA provides the hydrophobicity required preventing agglomeration, and by changing the amount of cross-linker either flexible (<20 wt % EGDM) or rigid nanocomposites could be fabricated. In our second method we fabricated thin polymer films using a radical free drop-casting process. Cellulose triacetate (CTA) was chosen as polymer because of its high transparency, hydrophobicity and thermal stability. It can be obtained from renewable resources such as wood or cotton. Moreover, it has already been found to be a suitable matrix for embedding CdSe/ZnS QDs [11–13]. The structure of CTA is shown in Figure 1. We used CdSe/CdS nanorods with two different sizes, longer rods with an aspect ratio of 6 and shorter bullet shaped rods with an aspect ratio of 3.

## Results and Discussion

### Nanorods

**Figure 2:**
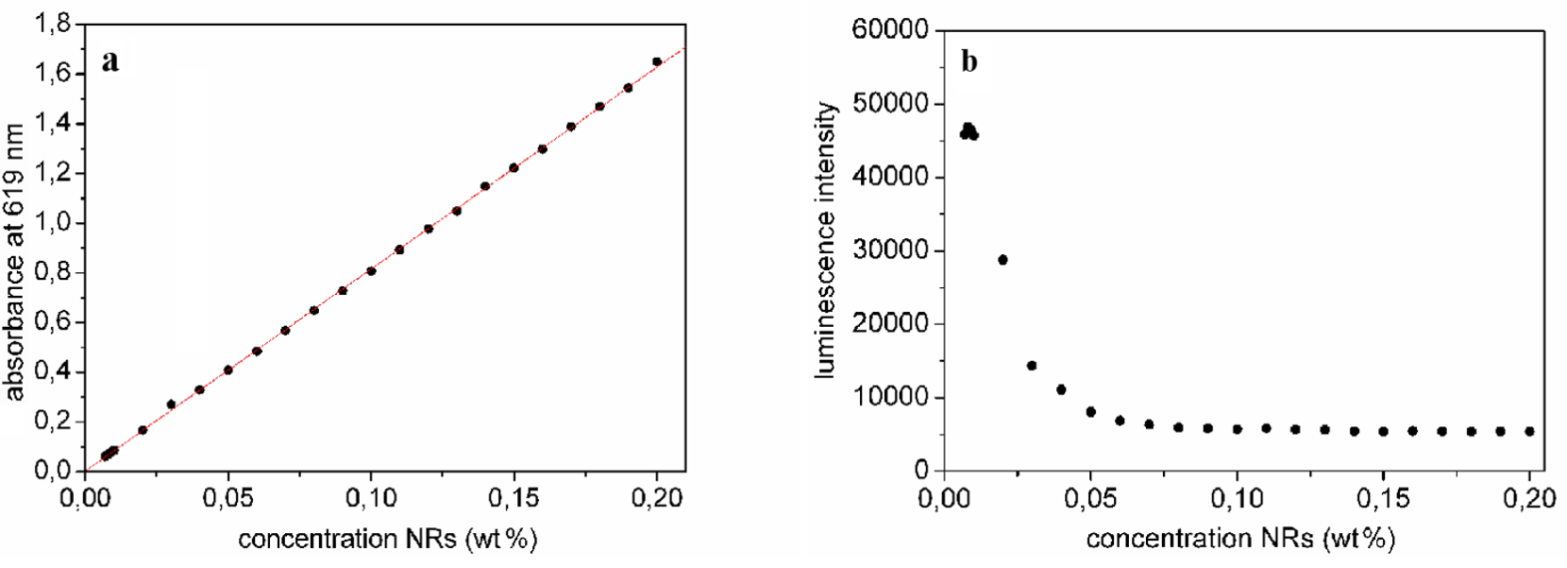
a) Absorption intensity of the first exciton peak (619 nm), b) luminescence intensity of the emission maxima (632 nm) as a function of concentration in chloroform solution for bullet shaped NRs (aspect ratio 3).

**Figure 3:**
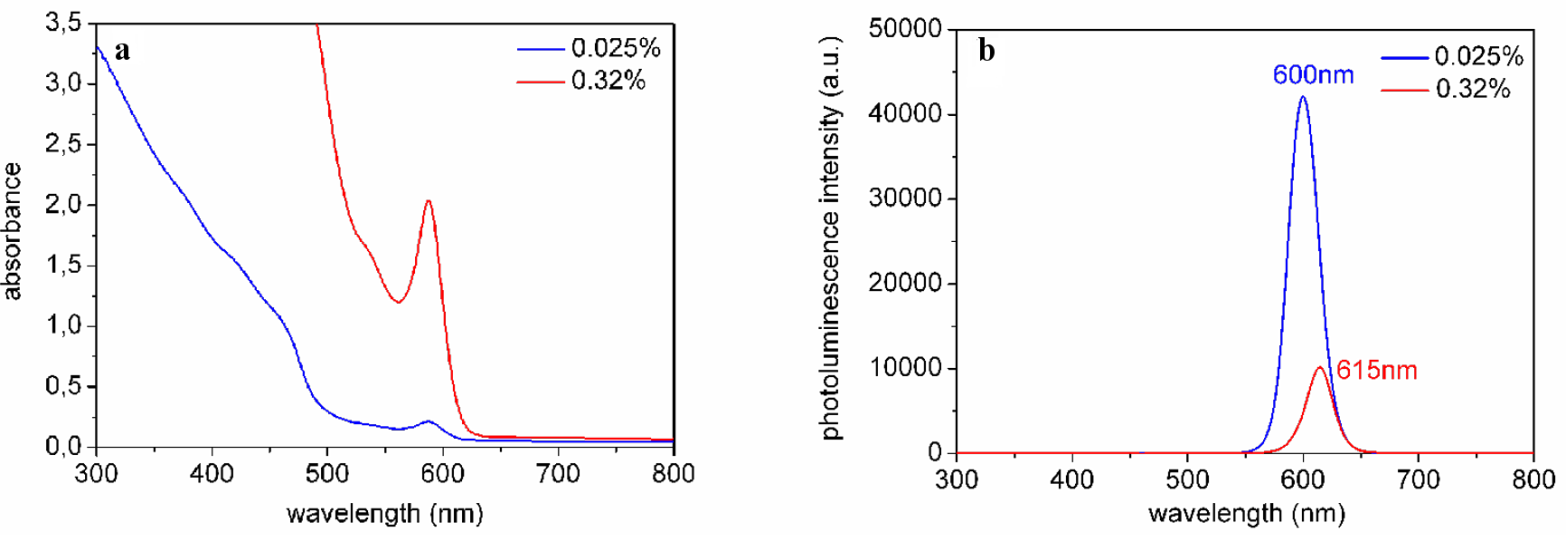
a) Absorption spectra and b) PL spectra of NRs (aspect ratio 6) in chloroform solution for different NR concentrations.

**Figure 4:**
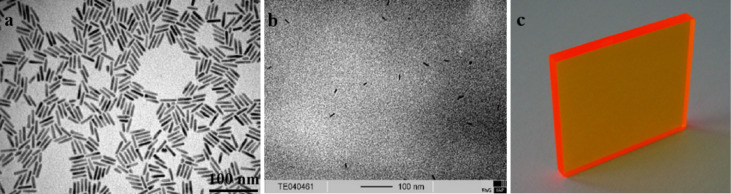
a) TEM image of CdSe/CdS nanorods (aspect ratio 6) b) in the NR/P(LMA-*co*-EGDM) composite and c) a photo of this nanocomposite (0.05 wt % NRs) showing bright luminescence already in daylight.

**Figure 5:**
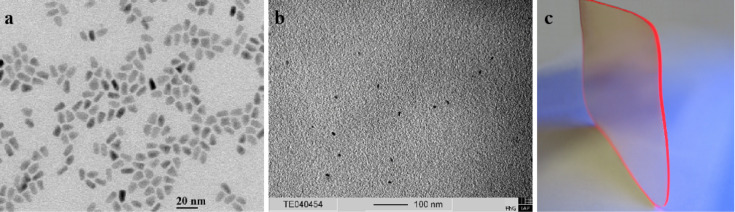
a) TEM image showing bullet shaped CdSe/CdS nanoparticles (aspect ratio 3), b) the NRs in the P(LMA-*co*-EGDM) matrix, and c) a photo of a NR/polymer composite (0.05 wt % NRs) illuminated with UV-radiation (365 nm).

Figure 2a shows the absorption maxima corresponding to the first exciton peaks for different aspect 3 NR concentrations (0.008–0.2 wt %) in chloroform. From the linear correlation between NR concentration and absorbance, we conclude that the Beer–Lambert law holds until at least 0.2 wt %. The emission maximum is at 632 nm, yielding a Stokes shift of 13 nm. The luminescence intensity (Figure 2b) has a maximum for a nanorod (aspect ratio 3) concentration of 0.008 wt %. Absorption and emission spectra for the aspect 6 NRs are shown in Figure 3a and 3b. At high concentrations, the emission is slightly shifted towards the red. As determined by TEM (Figure 4a, 5a), the longer rods had a diameter of 5 nm and a length of 30 nm (aspect ratio 6) and the shorter bullet shaped rods were 4 nm in diameter and 12 nm in length (aspect ratio 3).

### P(LMA-*co*-EGDM) nanocomposites

Clear nanocomposite plates without bubbles and smooth surfaces with sizes up to 8 × 8 × 0.5 cm^3^ were obtained (Figure 4c, Figure 5c). TEM measurements were performed on thin cryo-cut nanocomposite slices (770 × 540 × 60 nm^3^). The TEM image of a P(LMA-*co*-EGDM) nanocomposite slice containing 0.05 wt % aspect ratio 6 NRs is shown in Figure 4b: clearly, single NRs are homogeneously dispersed within the polymer matrix. We have counted a total number of 116 nanorods in 5 TEM images from different nanocomposite slices, which implies a NR concentration of ~1.5 µM. Figure 5b shows a TEM image of a nanocomposite containing 0.05 wt % NRs with an aspect ratio of 3. From a total number of 99 single distributed nanorods in 6 nanocomposite slices we deduce a NR concentration of ~1.1 µM within the P(LMA-*co*-EGDM) matrix. Thus, molar concentrations for NRs with aspect ratio 6 can be found using the scaling ~3 µM per 0.1 wt % whilst for NRs with aspect ratio 3 this is lower at ~2 µM per 0.1wt %.

**Figure 6:**
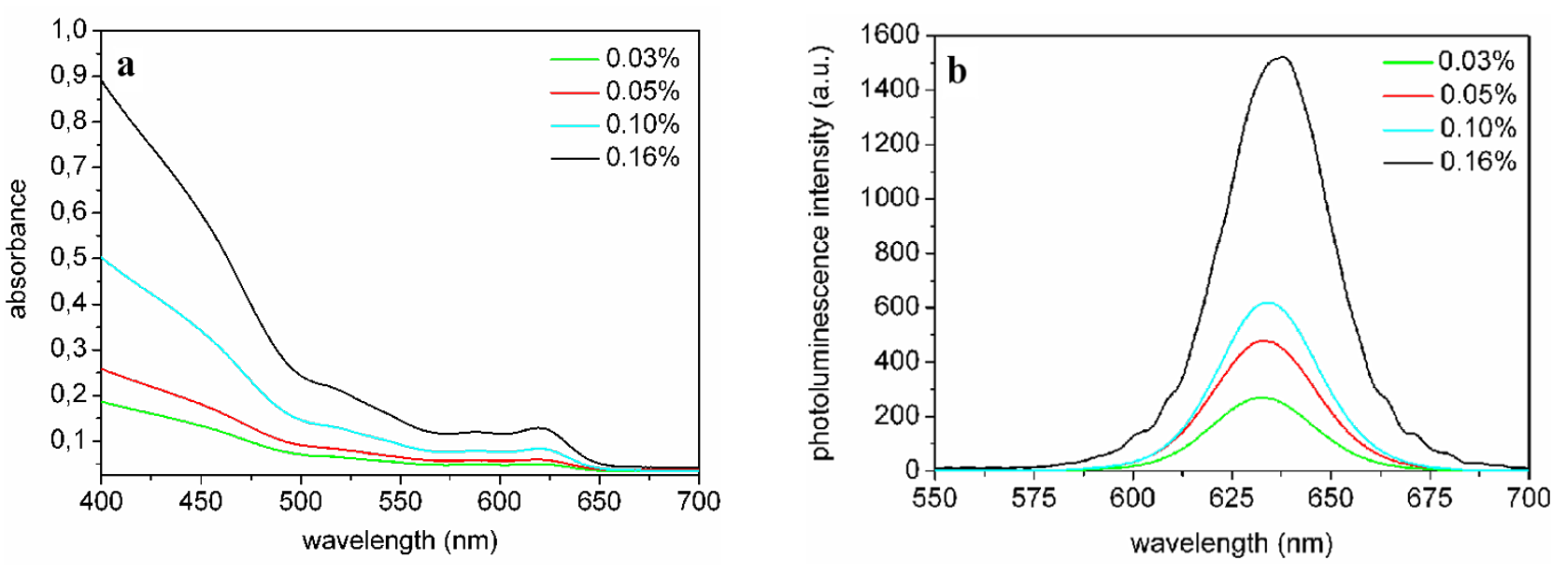
a) Absorption spectra and b) PL spectra of P(LMA-*co*-EGDM) nanocomposites containing NRs (aspect ratio 3) for different NR concentrations.

The absorption spectra of the P(LMA-*co*-EGDM) nanocomposites with NRs (aspect ratio 3) at different NR concentrations depicted in Figure 6a reveal strong absorption at shorter wavelengths (400–500 nm) and the first exciton peak at 619 nm. The nanorods are well dispersed in the polymer matrix, as shown by the lack of scattering in the absorption spectra at wavelengths much larger than that of the first exciton peak (Figure 6a). Figure 5c illustrates that we have fabricated highly transparent nanocomposites.

The PL spectra in Figure 6b show an increase of emission intensity as a function of NR concentration, and a small red shift of ~3 nm from 632 nm for low to 635 nm for high NR concentrations. From photoluminescence (PL) quantum efficiency (QE) measurements we find a high QE of 63% for nanocomposites containing nanorods with an aspect ratio of 3 for a concentration of 0.008 wt % (or 0.24 µM), see Table 1. For higher NR concentrations, the PL QE decreases to 37% for a concentration of 0.32 wt %. This can be readily explained by differences in the configurations of the PL and the PL QE measurements. The PL spectra were measured in reflection geometry, whereas the excitation light in the PL QE measurement system passes through the sample. In this case the emitted photons can be reabsorbed by other NRs, which causes PL quenching. Further, from Table 1 it can be inferred that the QE in solution for these NRs is somewhat lower than in the composite, suggesting a better stabilization in the polymer matrix.

**Table 1:** QE of P(LMA-co-EGDM) composites and chloroform solutions with aspect ratio 3 nanorods. The error in QE is 1%.

NR concentration (wt %)	PL QE nanocomposite (%)	PL QE chloroform solution (%)

0.008	63	62
0.03	52	43
0.05	49	41
0.10	40	-
0.16	43	40
0.32	37	-

**Figure 7:**
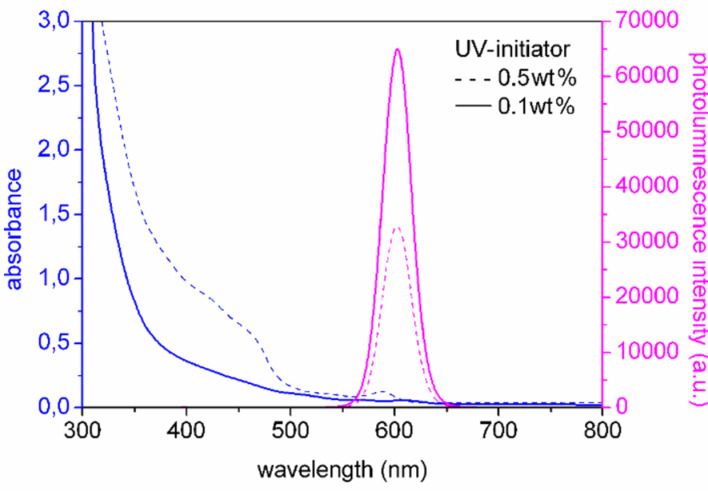
Absorption and PL spectra of NR/P(LMA-*co*-EGDM) composites containing 0.05 wt % NRs (aspect ratio 6) with low (0.1 wt %, solid line) and high (0.5 wt %, dotted line) UV-initiator concentration.

Figure 7 shows the effect of the concentration of the UV-initiator for nanocomposites with NRs with an aspect ratio of 6. A decrease in PL QE is observed for high UV-initiator concentration. It is therefore important to determine the lowest amount of UV-iniator that is required to obtain stable and fully polymerized plates. We found that, using only 0.1 wt % of UV-initiator, the best nanocomposite containing NRs with an aspect ratio of 6 reached a QE of 70% (for a NR concentration of 0.05 wt %). The PL QE in solution measured at 395 nm for an aspect ratio of 6 nanorods was 70%, which means no luminescence quenching occurred in the P(LMA-*co*-EGDM) nanocomposites. To our knowledge this is the highest luminescence quantum yield ever reached in a polymer nanocomposite containing luminescent nanorods.

**Figure 8:**
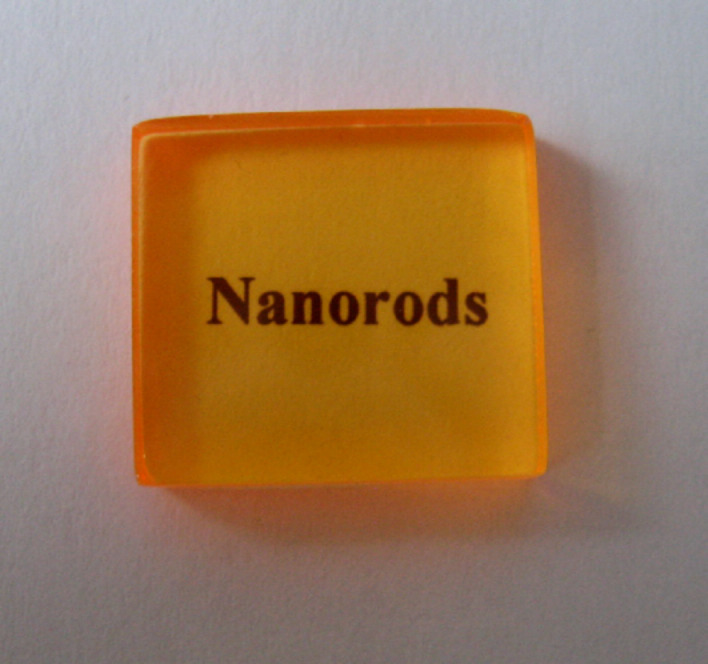
Photograph showing the high transparency of a 5 mm thick 0.05 wt % NR/P(LMA-*co*-EGDM) composite.

Finally, Figure 8 shows a photograph of a nanocomposite sample with NRs (aspect ratio 6) illustrating its high transparency. Nanocomposites with an aspect ratio of 6 NRs reach a maximum transmission of 92% in the spectral region between 630–800 nm. For an aspect ratio of 3, NRs polymer nanocomposites with a maximum transmission of 93% in the region between 700–800 nm have been fabricated.

### CTA nanocomposites

**Figure 9:**
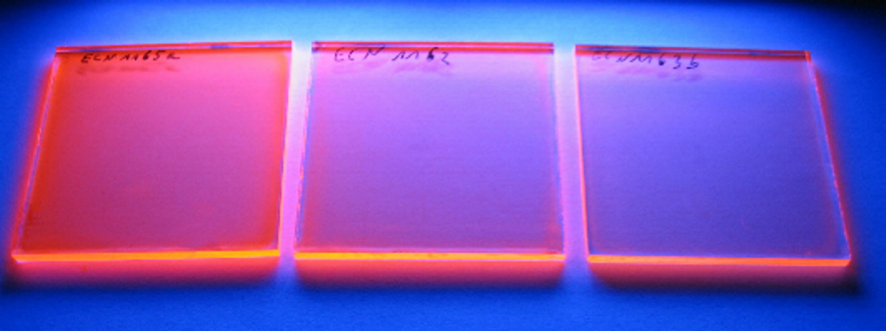
Photograph of CTA nanocomposite layers on glass substrates containing NRs (aspect ratio 6) illuminated with UV-radiation of 365 nm. The NR concentration is varied from left to right, i.e., 2 wt %, 1 wt %, and 0.5 wt %.

**Figure 10:**
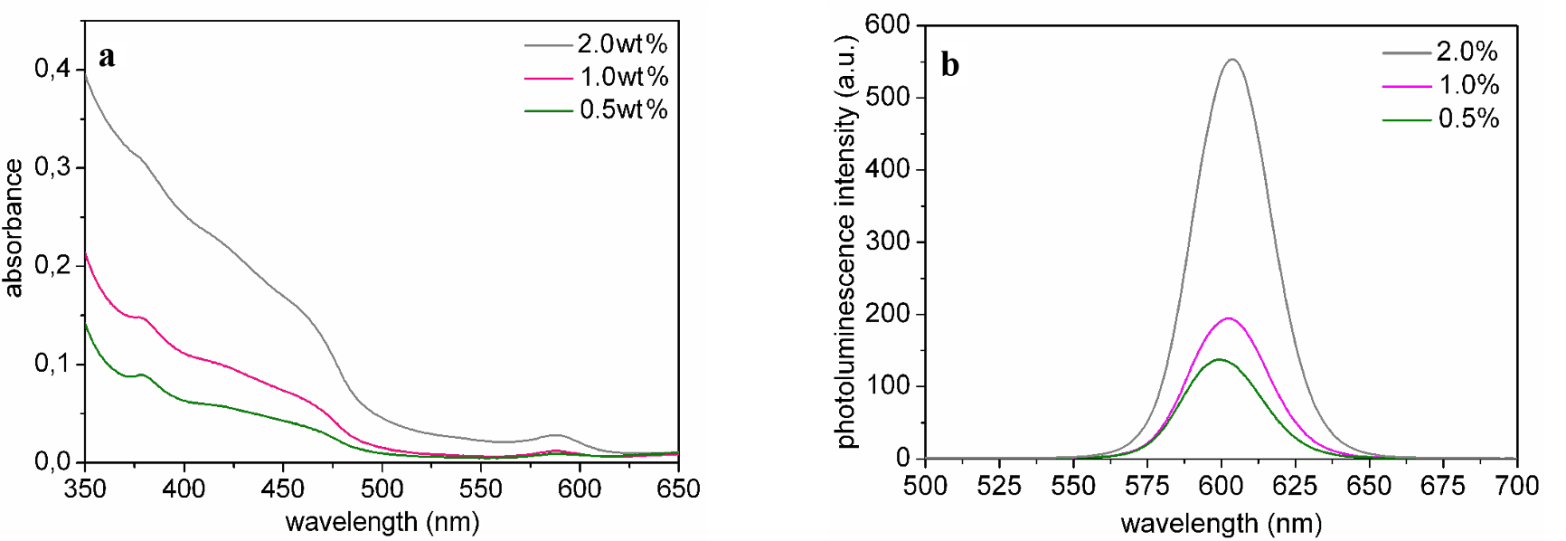
a) Absorption spectra and b) PL spectra of CTA nanocomposites containing NRs (aspect ratio 6) for different NR concentrations.

**Figure 11:**
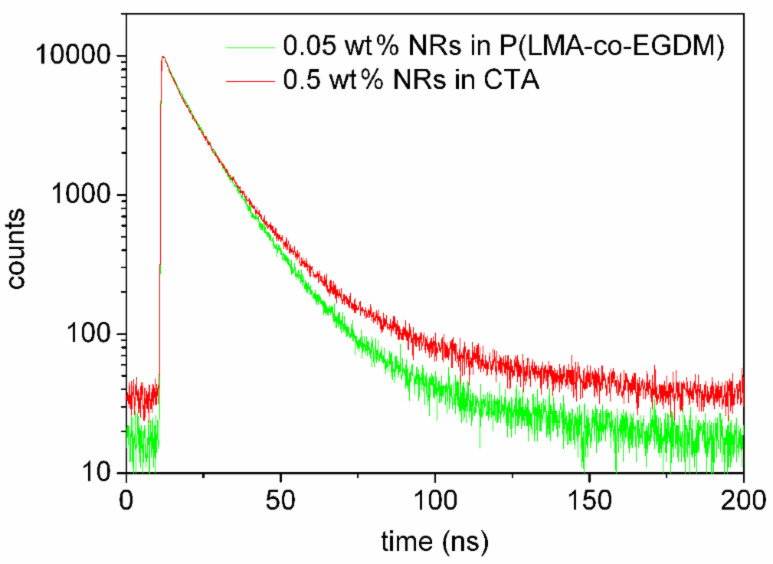
Decay curves of P(LMA-*co*-EGDM) and CTA nanocomposites containig aspect ratio 6 NRs.

The 9–15 µm thin CTA nanocomposite layers containing NRs (aspect ratio 6) on glass exhibit bright orange luminescence (see Figure 9). The absorption and emission spectra are shown in Figure 10. The QE ranges from 52% to 39% for NRs in CTA for 0.5 wt % and 2.0 wt %, respectively. Luminescence lifetime measurements revealed that dispersion of the nanorods in either PLMA or CTA did not influence the lifetime (Figure 11). Mono-exponential fits resulted in a lifetime of about 12 ns, while bi-exponential fits showed about 8 ns for the fast component and about 19 ns for the slow component.

**Figure 12:**
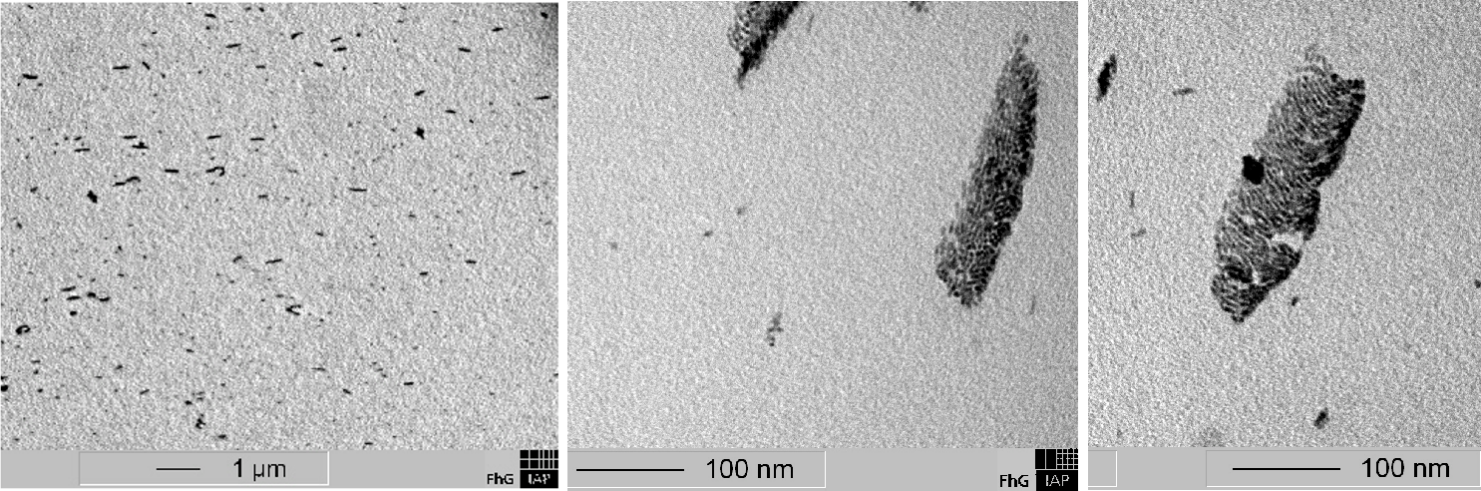
a) TEM images of a CTA nanocomposite layer with 2 wt % NRs (aspect ratio 6) and b) agglomerates with parallel and c) perpendicular NR orientation.

TEM images of the CTA nanocomposite layer containing 2 wt % NRs (aspect ratio 6), as shown in Figure 12, predominantly show rod-shaped agglomerates of hundreds of single NRs with diameters of about 60–80 nm and lengths of up to 300 nm, where the long axis is referred to as main agglomerate axis. From the TEM images, we deduce a concentration between 10–30 µM. Only a few single distributed nanorods can be seen. We found that the agglomerates are aligned within the CTA layer (Figure 12a), which can be explained by arrangement along the linear polymer chains and/or alignment in the casting direction (which was from left to right in this image). This is similar to spherical silver nanoparticles aligning in pearl-necklaces in the drawing direction when cast from nanoparticle polymer solutions, as observed by Dirix et al. [14]. In most of these NR agglomerates the NRs are aligned along the main agglomerate axis (see Figure 12b), but a few agglomerates with perpendicular NR orientation can be also found (Figure 12c). The distance between the single NRs within these agglomerates is ~2 nm.

## Conclusion

We have prepared highly transparent (>93%) luminescent (QE = 70%) nanocomposites from CdSe/CdS core/shell nanorods in PLMA plates and CTA thin layers. A high QE is observed at low nanorod concentrations (0.008 wt %) in PLMA, corresponding to a concentration of 0.24 µM. We found that a low as possible concentration of UV-initiator is required in order to obtain a high QE. In the case of CTA, we found that NRs are agglomerated.

## Experimental

The CdSe/CdS core/shell NRs used in this study were prepared following reported methods [13,15]. The nanoparticles were capped with different long alkyl chain ligands: the aspect ratio 3 nanorods were stabilized with a mixture of trioctylphosphineoxide (TOPO), trioctylphosphine (TOP) and hexadecylamine (HDA), whereas the aspect 6 nanorods were capped with a mixture of TOPO, hexylphosphonic acid (HPA) and octadecylphosphonic acid (ODPA). Various amounts of NRs (0.008–0.32 wt %) were dispersed in a monomer mixture of LMA (Fluka, 98%) with 20 wt % of the cross-linking agent EGDM (Fluka, ≥97%) and 0.1–0.5 wt % of the liquid UV-initiator Darocure^®^ 4265 (Ciba) by ultrasound treatment. The reaction mixtures were transferred into glass cuvettes and polymerized under a nitrogen atmosphere by illuminating the cuvettes from two sides with UV-A radiation (360 nm) for 15 min. The cuvettes consisted of two glass plates with an elastic distance holder (1 mm or 5 mm) between the plates, which were held together by steel clamps. The polymerized plates were taken out of the cuvettes and illuminated for additional 2 h to complete curing. The refractive index of the nanocomposites at 589 nm was determined with an Abbé refractometer to be 1.490 at 23 °C. The refractive index of the polymer itself (without NRs) is only slightly smaller (1.488) than that of the nanocomposites.

To prepare CTA nanocomposites, aspect ratio 6 NRs were dispersed in a mixture of CH_2_Cl_2_/CHCl_3_ (1:1) containing 2.5 wt % CTA (Eastman Chemical Company) by ultrasound treatment. The nanorod/polymer-solutions were drop-casted on 3 mm thick glass-substrates.

Absorption spectra were measured with a Perkin Elmer Lambda 950 UV–vis spectrometer. Emission spectra were recorded with a Perkin Elmer fluorescence spectrometer LS 50 B by exciting the samples at 395 nm. The PL QE of the P(LMA-*co*-EGDM) and CTA nanocomposites was measured at room temperature at the excitation wavelength of 395 nm with a Hamamatsu absolute PL quantum yield measurement system C9920-02, which uses an integrating sphere. Luminescence lifetime measurements were performed in a setup containing a FLS920 flourimeter (Edinburgh Instruments, Livingston, UK). A supercontinuum whitelight source (SC400-2, Fianium Ltd., Southampton, UK) was used as an excitation light source (rep. rate 5 MHz, λ_ex_ = 405 nm).
